# Japanese Legacy Cohorts: The Life Span Study Atomic Bomb Survivor Cohort and Survivors’ Offspring

**DOI:** 10.2188/jea.JE20170321

**Published:** 2018-04-05

**Authors:** Kotaro Ozasa, Eric J Grant, Kazunori Kodama

**Affiliations:** 1 Department of Epidemiology, Radiation Effects Research Foundation, Hiroshima, Japan; 2 Associate Chief of Research, Radiation Effects Research Foundation, Hiroshima, Japan; 3 Executive Director, Radiation Effects Research Foundation, Hiroshima, Japan

**Keywords:** atomic bomb survivors, radiation, late health effects, transgenerational effects

## Abstract

Cohorts of atomic bomb survivors—including those exposed *in utero*—and children conceived after parental exposure were established to investigate late health effects of atomic bomb radiation and its transgenerational effects by the Atomic Bomb Casualty Commission (ABCC) in the 1950s. ABCC was reorganized to the Radiation Effects Research Foundation (RERF) in 1975, and all work has been continued at RERF. The Life Span Study, the cohort of survivors, consists of about 120,000 subjects and has been followed since 1950. Cohorts of *in utero* survivors and the survivors’ children include about 3,600 and 77,000 subjects, respectively, and have been followed since 1945. Atomic bomb radiation dose was estimated for each subject based on location at the time of the bombing and shielding conditions from exposure, which were obtained through enormous efforts of investigators and cooperation of subjects. Outcomes include vital status, cause of death, and cancer incidence. In addition, sub-cohorts of these three cohorts were constructed to examine clinical features of late health effects, and the subjects have been invited to periodic health examinations at clinics of ABCC and RERF. They were also asked to donate biosamples for biomedical investigations. Epidemiological studies have observed increased radiation risks for malignant diseases among survivors, including those exposed *in utero*, and possible risks for some non-cancer diseases. In children of survivors, no increased risks due to parental exposure to radiation have been observed for malignancies or other diseases, but investigations are continuing, as these cohorts are still relatively young.

## ORIGIN OF THE COHORT

In August 1945, two atomic bombs were exploded over the cities of Hiroshima and Nagasaki and generated shock waves, thermal energy, and ionizing radiation. People suffered from physical injuries and acute radiation syndromes, and late health effects continue to this day. Local governments reported that approximately 140,000 people in Hiroshima and 74,000 in Nagasaki died before the end of 1945.^1^^,^^2^ Medical aspects of physical injuries and acute radiation syndrome have been investigated in the survivors since shortly after the bombings. Studies of possible genetic effects in the offspring, including birth defects, still birth, neonatal death, and the sex ratio of newborns, were initiated in the late 1940s to early 1960s.^3^^,^^4^

In the earliest period after the bombings, the medical conditions of the survivors and the environmental radioactive levels were intensively investigated by scientists from Japanese universities, the United States (US)-Japan Joint Commission, and others.^4^ In 1947, the Atomic Bomb Casualty Commission (ABCC) was established in Hiroshima by the US National Academy of Sciences and the National Research Council, and another laboratory of ABCC was opened in Nagasaki in 1948. In the same year, a branch of the Japanese National Institute of Health of the Ministry of Health and Welfare was opened in the facility of ABCC, so that ABCC was positioned as a cooperative research agency by the two nations. Funds were provided to the ABCC by the US Atomic Energy Commission, the Japanese National Institute of Health, and the US Public Health Service. In the early 1970s, the US Public Health Service was replaced by the US National Cancer Institute, US National Heart and Lung Institute, and US Environmental Protection Agency. In 1975, ABCC was reorganized to the Radiation Effects Research Foundation (RERF) under Japanese civil law. RERF is administered bi-nationally by a board of Japanese and US councilors and is funded by both Japanese and US governments. All ABCC research activities were carried on by RERF.^3^^,^^5^

Individual projects for medical investigations in the late 1940s to early 1950s were based on ad hoc definitions of study subjects and radiation exposure, sometimes resulting in inconsistent results. In 1955, the Francis Committee, an ad hoc committee to evaluate ABCC research programs, recommended a “unified central program” with defined exposure classes (ie, proximal and distal exposed and non-exposed, as described below) and a fixed population base for the program. An epidemiological detection network and clinical detection program were also recommended.^6^ Thus, the Life Span Study (LSS), a cohort study of atomic bomb survivors, was initiated. Subsequently, cohorts of those who were exposed in the mother’s womb (*in utero*) and children of survivors (F_1_) were constructed.^3^^,^^5^ Subsets of each of those cohorts were further selected for clinical investigations.

## SCOPE AND FEATURE

The purpose of ABCC-RERF’s cohort studies is to investigate the late health effects of exposure to atomic bomb radiation among the atomic bomb survivors, including those exposed *in utero*, as well as hereditary or transgenerational effects among children conceived after parental exposure. Late health effect outcomes include cancer and other chronic diseases that may be caused by radiation exposure. According to RERF’s Articles of Incorporation, RERF’s objective is to “conduct research and studies for peaceful purposes on medical effects of radiation and associated diseases in humans, with a view to contributing to maintenance of the health and welfare of the atomic bomb survivors and to enhancement of the health of all humankind.”^7^ As the two cities were non-selectively bombed, cohort subjects are representative of the general population of the cities. Individual radiation doses of the subjects ranged from zero to the highest survivable levels, as some survived acute radiation syndromes after exposure. Therefore, these cohorts are singularly unique in human history.

## BUILDING AND MAINTAINING THE COHORT

### Cohorts

**Figure 1.**
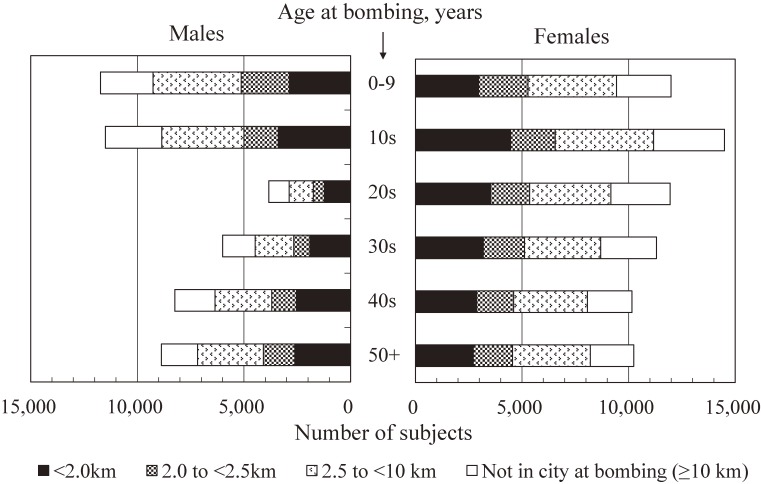
Distribution of LSS subjects by sex, age at bombings, and distance from hypocenters

The National Census of Japan in 1950 included a supplementary survey for atomic bomb survivors that identified approximately 284,000 survivors throughout Japan. Among them, about 195,000 survivors were residing in Hiroshima or Nagasaki at the time of the census. ABCC personnel contacted more than 99% of them to collect basic information about survivors’ situation at the time of the bombings (eg, location and simple shielding information). Indeed, such information was already collected from a considerable proportion of the target population in previous surveys since the late 1940s. The LSS cohort included four groups: (1) all survivors who were located within 2 km of either hypocenter at the time of the bombings (“inner proximal”), (2) all survivors at 2 to <2.5 km (“outer proximal”), (3) survivors at 2.5 to <10 km who were sex- and age-matched to the inner proximal survivors (“distal”), and (4) people who were not in either city (ie, >10 km of the hypocenters) at the time of the bombings and sex- and age-matched to the inner proximal survivors (“not-in-city”). Because the records of residents other than respondents to the supplementary survey at the 1950 Census were discarded at the time of sampling, group (4) was selected from other rosters provided by Hiroshima and Nagasaki city offices and ABCC surveys in order to represent the population at the 1950 Census in both cities. Initial subjects of groups (1) and (2) were limited to those whose permanent domicile (*honseki*) were in Hiroshima or Nagasaki cities. Later, those with *honseki* in other areas were included in 1968, and the remaining distal survivors in Nagasaki were included in 1980.^3^ So, the initial design of sex- and age-matched groups was modified but, starting in the mid-1960s, individual dose estimates became available, obviating the need for distance-based categories. With these additions, the LSS cohort consists of a total of 120,321 subjects (increased from an initial selection of roughly 100,000 subjects), including 34,363 in group (1), 19,959 in group (2), 39,419 in group (3) (Note: this group includes 14 subjects who were identified to have been located slightly beyond 10 km of the hypocenters by later detailed surveys), and 26,580 in group (4), according to the information used for DS02R1 individual doses.^8^ Demographically, the cohort consists of 82,214 from Hiroshima and 38,107 from Nagasaki (50,175 males and 70,146 females). There was a general lack of men of age in the 20s to 30s, who were thought to be out of the cities due to military duties and related jobs (Figure 1). All cohort members have been followed since 1950. As the cohort was constructed in the late 1950s, the LSS is partially a retrospective cohort. But, essential baseline information, such as location and simple shielding conditions at the time of the bombing, was collected from most of the subjects around 1950. So, it is thought that selection bias and recall bias were minimized with retrospective construction of the cohort back to 1950, given the long follow-up period thereafter.^9^ Another potential selection bias due to survival of the subjects from 1945 to 1950 is discussed later. From the LSS, 24,358 subjects were invited to the biennial health examination program called the Adult Health Study (AHS) at ABCC clinics in Hiroshima and Nagasaki since 1958.^3^

Persons who were exposed *in utero* to atomic bomb radiation were identified using birth reports and other information. Their mother’s condition at the time of the bombings was investigated based on the information of ABCC. Study subjects were selected based on categories of distance from the hypocenters. Some subjects were added from a supplementary survey at the 1960 National Census. The *in utero* cohort consists of 3,638 subjects and has been followed since 1945 or 1960. Some 1,021 cohort members have been invited to biennial health examinations since 1976.^3^

Parents of children conceived after the atomic bombings and born in Hiroshima or Nagasaki were interviewed regarding their exposure to the atomic bombings via interview and census records. A children’s cohort (the “F_1_”) was selected using stratified sampling of parental exposure status and matching for sex and year of birth across the strata. Children born to parents who were both “not-in-city” were also included. Later, other children born to members of the LSS were also added to the cohort. Detailed recruitment procedures were complicated and are described elsewhere.^3^^,^^10^ The F_1_ cohort consists of 76,814 singleton children and has been followed since 1946. A health examination program for selected F_1_ members began in 2002, when they were entering the age range at which the occurrence of cancer and multifactorial diseases increases. A total of 11,951 subjects participated in the health examination during 2002 to 2006 and are being followed through repeated exams every four years.^3^

As of the end of 2013, about 30%, 35%, and 90% of LSS, *in utero*, and F_1_ cohort subjects were alive, respectively.

### Exposures

**Figure 2.**
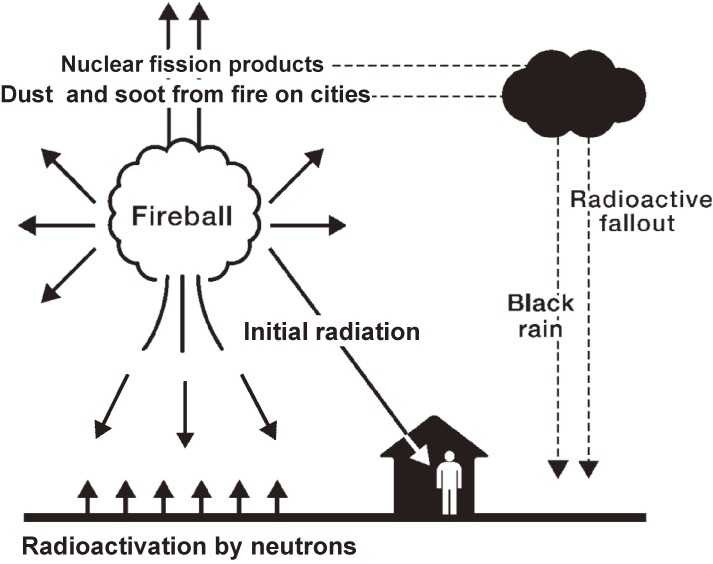
Scheme of radiation released from atomic bombs. Atomic bomb radiation consisted of initial radiation and residual radiation. Initial radiation was emitted directly from the fireball. Gamma rays and neutrons reached people on the ground. Residual radiation was released from activated materials by neutrons and fission products. Fission products went up to high atmospheric layers with rising air from the fireball, then cooled and condensed. Some fallout fell down with rain just after the bombings. The rain often contained dust and soot from the fires in the cities and so was called “black rain^11^”. (Modified from ref. ^11^)

Radiation from atomic bomb explosions is classified into initial radiation and residual radiation. Initial radiation was emitted directly from nuclear fission and fission products in fireballs, resulting in exposures to gamma rays and neutrons on the ground. Residual radiation was released from neutron-induced radioactive materials in the environment and fission products contained in fallout. Such fallout often fell down with rain just after the bombings, as fission products cooled as they rose to high atmospheric layers. The rain often contained dust and soot from the remains of the cities (Figure 2).^3^^,^^11^

The first systematic estimation of atomic bomb radiation doses was developed in the 1950s and designated T57D (“T” represents “tentative” and the number indicates the year of development),^12^ but it was not officially used in ABCC studies.^5^ The T65D system was officially used for individual dose estimates of survivors.^13^ DS86 was the first comprehensive dosimetry system developed in collaboration by US and Japanese scientists.^14^ DS02 was developed to correct remaining inconsistencies in DS86.^15^^,^^16^ Since DS86, individual doses of initial radiation have been estimated based on (1) amount of radiation emitted from the bombs, (2) transfer from fireball to persons, and (3) shielding conditions of persons. The factors (1) and (2) were estimated using nuclear physics, demonstration experiments at nuclear test fields in the US, and measurements of elements in metal and concrete exposed to atomic bomb radiation in Hiroshima and Nagasaki. Detailed shielding conditions of about 20,000 proximal survivors in the LSS were investigated using interview surveys in which location at the time of the bombing was identified on a map and surrounding buildings were drawn to evaluate shielding; in addition, if the survivors was inside a house, the house layout was drawn and location and posture of the survivor was identified. Based on this information, individual neutron and gamma doses for 15 organs were estimated. “Weighted absorbed organ dose,” in which neutron dose is weighted by a relative biological effectiveness factor (ie, neutron dose × 10 + gamma dose), has been used to evaluate health effects. Since detailed surveys were not conducted for distal survivors, their individual dose was estimated based on the information from the early contacts (location at the time of the bombings and simple shielding information), as well as average transparency coefficients for Japanese wooden houses and other results that were obtained in the above-detailed investigations.^15^^,^^16^ Recently, individual shielding information and location were reviewed, and individual radiation doses subsequently revised as DS02R1 are currently used at RERF.^8^

Unshielded weighted radiation dose was estimated to be about 7 Gy and 10 Gy at 1 km from the hypocenters in Hiroshima and Nagasaki, respectively, but only 13 mGy and 23 mGy, respectively, at 2.5 km.^15^ The distribution of individual dose estimates of LSS subjects is shown in Table 1.^8^ The total number of LSS cohort members who were exposed to initial radiation doses of 1 Gy or higher according to DS02R1 was about 2,200, whereas almost half were exposed to around 5 mGy or less. Most subjects with unknown dose were located in large concrete buildings or bomb shelters in proximal areas. Due to these complex shielding conditions their individual doses could not be estimated.

**Table 1.** Distribution of LSS subjects by radiation dose

Dose^a^ (Gy)	Number of subjects
Dose known	86,720
<5	45.0%
5–99	34.2
100–499	14.3
500–999	3.9
1,000–1,999	1.9
≥2,000	0.6
Dose unknown	7,021
Not in city at bombings	26,580

Total	120,321

^a^Weighted absorbed colon dose in DS02R1.

Sum of presented percentages is not 100% because of rounding.

**Figure 3.**
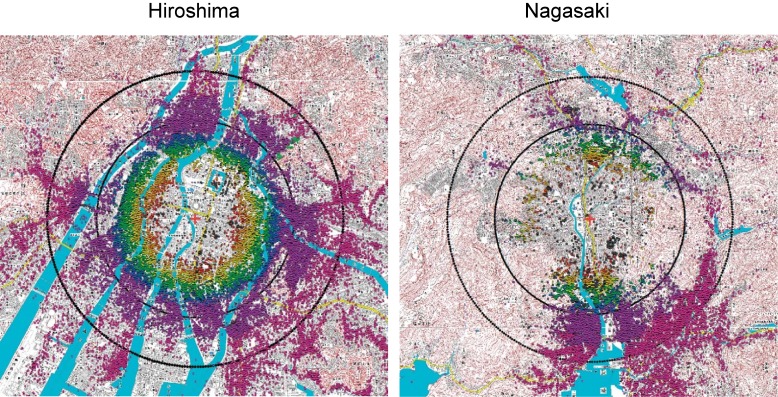
Spatial distribution of LSS subjects with grouped radiation dose estimates. The points on the maps indicate the locations at exposure to atomic bomb explosion of LSS subjects. Color key starting with bottom layer: pink, <5 mGy weighted colon dose; purple, 5 to 100; blue, 100 to 200; green, 200 to 500; yellow, 500 to 1,000; orange, 1,000 to 2,000; red, >2,000; dark gray, unknown dose. Black circles at 2 and 3 km from the hypocenter.^8^

The spatial distribution of subjects within dose categories is shown in Figure 3. Those who were exposed to 1 Gy or higher were within a relatively narrow distance range from the hypocenters (between concentric circles at about 0.7 and 1.5 km) and are, therefore, assumed to be relatively homogenous in socioeconomic and lifestyle factors, with no selection factors for exposure. Therefore, there should be virtually no potential for bias or confounding by other major risk factors of cancer and other lifestyle-related diseases. Late health effects are assumed to develop based on subclinical injuries of cells; for example, DNA injuries that might induce a later cancer may not necessarily indicate immediate clinical manifestations. The dose of atomic bomb radiation to bone marrow at which half of exposed people should die within 60 days (ie “LD_50/60_”) is estimated to be 2.9 to 3.3 Gy in DS02.^3^^,^^17^ The observed radiation dose-response using an excess relative risk model was usually linear for solid cancers in the LSS (described later), which is consistent with the expectation that one additional injury to a tumor suppressor gene could induce a loss of heterogeneity. Therefore, survival between 1945 and 1950, when the Japanese national census occurred (and was the basis for constructing the LSS cohort), should not cause a large selection bias at radiation exposures less than about 4 Gy of shielded kerma (roughly equivalent to bone marrow dose of 2.7 Gy in the LSS). All these points taken together indicate that the strong radiation risks observed at 1 Gy or higher are thought to be causal. In contrast, distal survivors were distributed across wide areas, so risk factors for cancer and other diseases might be related to residential districts, which may also correlate with distance from the hypocenters, and hence, individual radiation dose. Therefore, caution must be used when interpreting radiation effects at low dose levels due to their vulnerability to possible confounding by lifestyle-related risk factors.

As for residual radiation, the amount of neutron-induced radiation is theoretically based on the number and strength of neutrons that were emitted from the explosions and the type and distance of elements located in soil and buildings. Calculations indicate that a person who stood 200 meters from the hypocenters for 12 hours the day after the bombings would be exposed to about 82 mGy and 12 mGy in Hiroshima and Nagasaki, respectively, from neutron-induced radiation.^3^ There were some hot spots with high ground radioactivity due to radioactive fallout in both cities based on ground surveys conducted within several months after the bombings. Radiation doses from those sources were estimated to be between 6 to 24 mGy and 120 to 240 mGy at hot spots in Hiroshima and Nagasaki, respectively.^14^ In order to estimate individual doses from residual radiation, information on route and time of evacuation after the bombings for each survivor and radioactivity on the ground must be known. As such data were not systematically collected, RERF studies generally ignore these doses. This decision is justifiable due to the low average expected levels of residual radiation in Hiroshima and the small number of people affected in a remote, high radioactive area in Nagasaki (roughly a few hundred persons). Therefore, exposure to residual radiation should not have a large influence on radiation risk estimates based on the information of high initial radiation doses.^3^ Follow-up studies of mortality and incidence of cancer and prevalence of acute symptoms among those exposed to fallout rain did not indicate strong health effects according to exposure to residual radiation as a group, although adverse health effects among some distally-exposed individuals have been reported.^18^^,^^19^

Lifestyle risk factors for outcomes such as cancer and other diseases were not collected in the early interview surveys, so mail surveys using self-administered questionnaires have been conducted five times (and an additional three times among the AHS subjects) to collect information on smoking and drinking habits and health conditions.^3^

### Outcomes

Vital status of RERF cohort members has been periodically checked via access to Japan’s official family registry system (*koseki*) since 1950. If a person has died, a copy of the death certificate is reviewed and the cause of death is recorded. Cancer incidence information has been collected via linkage with cancer registries. Until 2015, RERF linked with local cancer registries in Hiroshima and Nagasaki; since 2016, linkage is performed via Japan’s recently initiated National Cancer Registry. Local cancer registries in Hiroshima and Nagasaki were among the earliest to be established in Japan and have long histories. The Tumor Registry of Hiroshima City Medical Association began in 1957 and is currently known as the Hiroshima City Cancer Registry. The Hiroshima Prefecture Cancer Registry began in 2002. The Tumor Registry of Nagasaki City Medical Association began in 1958 and was merged with the Nagasaki Prefecture Cancer Registry, which began in 1985. In addition, pathological tissue registries were established in 1973 and 1974 in Hiroshima and Nagasaki, respectively, to collect precise information of tumor diagnosis in local hospitals by prefectural medical associations in cooperation with local pathologists. RERF has contracted with these associations and has confirmed the high quality of the local cancer registries by merging information from these sources. In regard to hematopoietic malignancies, the Leukemia Registry was initiated by ABCC in 1946 but ceased activities in the early 1990s, when it was no longer deemed necessary. Currently, information on leukemia incidence is registered through local cancer registries and pathological tissue registries.

In the periodic health examinations of clinical sub-cohorts (AHS and others), routine medical examinations for lifestyle-related and other diseases have been carried out, and the participants have been asked to donate blood and urine for further scientific investigation. These biosamples have been frozen and stored.

Autopsies of deceased survivors and other people in Hiroshima and Nagasaki were intensively carried out by ABCC since the 1940s. But, with a general tendency towards decreased number of autopsies performed, the autopsy program at ABCC-RERF terminated in the late 1980s.^5^ As survivors were medically treated in local hospitals, pathological specimens resected through surgical operation are stored at the hospitals. A number of special cancer studies—in which these tissue samples were gathered and reviewed—have been conducted in collaboration with local pathologists.^3^

## MAJOR PUBLICATIONS

### LSS and AHS

Results of the LSS follow-up have been published in a number of periodic reports, as well as comprehensive reports called “LSS Reports”^20^^–^^38^ (Note: the number of reports differed between the journal papers and the ABCC-RERF Technical Reports from Reports 4 to 11,^23^^–^^34^ but this was resolved as of Report 12^35^^,^^36^ when the Technical Reports were abolished). The first two reports, “LSS Reports 1 and 2”,^20^^,^^21^ documented the construction of LSS cohort and the results from a partial cohort that had already been constructed at the time of those reports. Based on group comparisons classified according to distance from the hypocenters, an excess occurrence of leukemia was observed in proximal survivors, but excess occurrences of solid cancer and other diseases were unclear. Results with all subjects (about 100,000 at that time) observed during 1950–60 were first reported in the third report. Proximal survivors showed higher mortality from all causes, all diseases, leukemia, and other malignant neoplasms when compared to distal survivors.^22^ T65D was first used for verification of radiation exposure in survivors in 1971.^24^ Radiation effects from the atomic bombs gradually became clearer as outcome data accumulated. Individual dose estimates from DS86 were first used for risk analyses in 1989.^32^ The risk measure excess relative risk (ERR), which is the proportion of excess rate due to radiation exposure divided by background rate (equivalent to relative risk minus one), was first used in “Report 12”.^35^^,^^36^ ERR has both additive and relative aspects of risk and is a convenient way to express the nature of radiation risk, which is assumed to be proportional to the number of cells injured by radiation exposure. For example, an ERR of 0.5 indicates a 50% increase above the background rate of outcomes. Thereafter, analyses of dose-response modeling have been extended to include effect modification by age at exposure, attained age, and other factors. DS02 was used in “Report 14”.^38^ A comprehensive report on radiation risk of incidence of malignancies reported by local cancer registries was first published in 1994^39^^–^^41^ and has been updated for leukemia and solid cancer in separate reports.^42^^–^^44^

**Figure 4.**
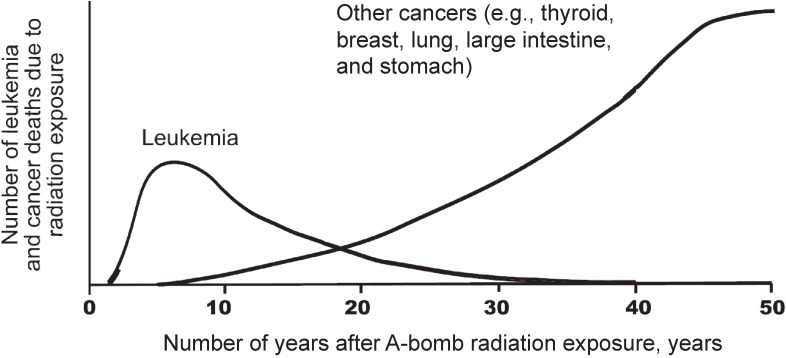
Trend of excess cases of leukemia and cancer due to atomic bomb radiation exposure by year since exposure (model schema). Excess of leukemia increased shortly after the bombings then decreased while detection of solid cancers increased about 10 years after the bombings and has continued thereafter.^66^

**Figure 5.**
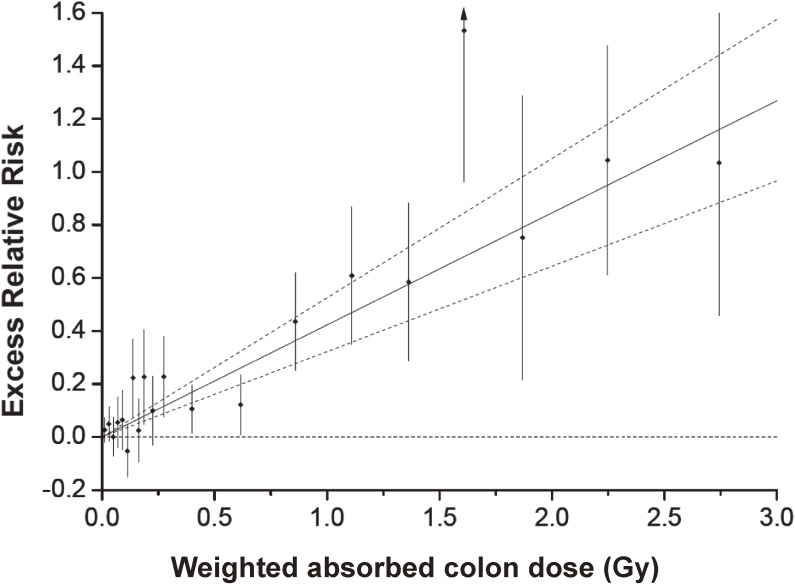
Excess relative risk (ERR) for all solid cancers in relation to radiation exposure. The black circles represent ERR and 95% confidence intervals (CIs) for dose categories. The solid line indicates the best fit linear model over the full range with 95% CIs (dotted lines). ERR/Gy = 0.42 (95% CI, 0.32–0.53) for the gender-averaged risk estimates for a person aged 70 years who was exposed at age 30 years based on the model with effect modification by sex, age at exposure, and attained age. The ERR was significant over the dose range of 0 to 0.20 Gy or higher. The estimated threshold dose was 0.0 Gy with upper 95% confidence limit was 0.15 Gy.^38^

Based on those reports, the late health effects of atomic bomb radiation can be summarized as follows. An excess of leukemia cases began to appear about 2 years after the bombings, peaked at about 6 to 8 years after exposure, and then rapidly decreased, but not to zero, as a slight increase has persisted for more than 5 decades (Figure 4). The risk was higher among those exposed at young ages. The shape of the ERR dose response for acute myeloid leukemia was non-linear, a concave curve, while those for acute lymphocytic and chronic myeloid leukemia were mostly linear.^43^ Risk of solid cancers increased around 10 years after the bombing and continue to be elevated today. The ERR of all solid cancer increases linearly with radiation dose, with a relative increase of around 40 to 50% per Gy for both mortality and incidence for a person at age 70 years who was exposed at age 30.^37^^,^^38^^,^^42^^,^^44^ These estimates are not confounded by smoking.^44^ The risk is increased significantly at radiation dose levels of about 0.1 to 0.2 Gy or higher, and the modeled linear dose response does not strongly indicate a threshold (95% confidence intervals of 0.08 to 0.15 Gy).^38^^,^^42^ Figure 5 shows a typical dose-response shape.^42^ The latest analysis of all solid cancer incidence indicated a dose response with significant concave curvature in men, and the reasons for this finding are under investigation.^44^ Radiation risk of cancer varies between organs. Presently, risks of cancers of the bladder, female breast, lung, brain, thyroid gland, colon, esophagus, ovary, stomach, liver, and skin (excluding melanoma) have apparently increased significantly, but those of the pancreas, rectum, uterus, prostate, and kidney parenchyma have not. Relative risks of radiation were higher in survivors exposed at young ages but have decreased with attained age, as background levels increased. Excess absolute rates were also higher in those exposed at young ages and have continued to increase with attained age.^38^^,^^42^

Radiation risks of malignant neoplasms at high dose levels, such as 1 Gy or higher, are considered to be causative in the LSS cohort, whereas risks at low dose levels are still equivocal, especially at doses under 100 mGy. At low doses, the effect is predicted to be small, which limits statistical power to detect the effects. In addition, other factors become more problematic, including confounding by lifestyle factors that may be associated with residential areas (associated with distance from the hypocenters). Other issues include possible interactions of radiation with other risk factors and unmeasured exposures to residual radiation and/or medical radiation.

Risk of non-cancer diseases has been evaluated using mortality statistics in the LSS and health examinations in the AHS. Periodic LSS Reports have shown small significant increases in ERR for circulatory, respiratory, and digestive diseases.^38^ Although heart disease and stroke showed significant increases in ERR as a whole, ERR estimates for their subtypes (ie, ischemic heart disease, cerebral hemorrhage, or infarction) were not increased, but heart failure and unspecified types of stroke did show increases.^45^ Similar findings were observed in detailed analyses of heart disease.^46^ For respiratory disease, a linear increase in ERR was observed for pneumonia after the 1980s, a period when pneumonia transitioned from an acute affliction to a terminal stage disease in the elderly.^47^ Diseases that were associated with increased radiation risks could often be described as ill-defined diagnoses (eg, heart failure and pneumonia) that were common in the elderly, so it is conceivable that apparent increased risks for such diseases might be partially explained by misclassification of the underlying cause of death as designated on death certificates, especially potentially hidden malignant diseases.

Results from the AHS have also been reported periodically. The first several reports outlined the results of specific examination cycles; comprehensive reports were published in 1993 and 2004.^48^^,^^49^ In addition, there have been many publications of studies targeting specific diseases, including circulatory, renal, hepatic, and thyroidal disorders, cataract, and others based on special information and clinical examinations. Donated biosamples have been used for many studies of the pathophysiology of radiation-related diseases, including carcinogenesis and immunosenescence.^3^

### Cohort of *in utero* exposed

Impaired physical and mental development among children exposed *in utero* was reported.^3^ Associations between *in utero* exposure and excess cancer mortality or incidence were not observed in the early periods, probably due to the small number of outcomes.^50^^–^^53^ After accumulation of sufficient data that accompanied the aging of the cohort, associations have been detected,^54^^–^^57^ and the magnitude of the risks were comparable to those observed in survivors who were exposed at young ages.^56^^,^^57^ However, it is still unknown which period of gestation has the highest sensitivity to radiation. Excess occurrences of leukemia have not been observed, probably because the cohort is small and leukemia is a rare outcome.

### Children of survivors (F_1_)

Genetic studies have indicated no significant associations between parental exposure to atomic bomb radiation and the frequency of genetic abnormalities among children conceived after the exposure.^3^ Epidemiological follow-up has indicated no increased risk of cancer or non-cancer disease mortality or cancer incidence associated with parental radiation dose.^10^^,^^58^^–^^62^ From the health examination program, prevalence of multifactorial diseases, including hypertension, hypercholesterolemia, diabetes mellitus, angina pectoris, myocardial infarction, and stroke has, so far, not been associated with parental radiation exposure.^63^ As the F_1_ cohort is still relatively young, continued follow-up is necessary.

## HISTORICAL IMPACT ON GLOBAL OR LOCAL HEALTH

The results of these cohort studies have been used as the most reliable source of evidence to estimate the long-term health risks in radiation-exposed humans. They have been used to justify medical and welfare subsidies for the survivors and their children by the Japanese government and also used for scientific evaluation of radiation effects by many international organizations. Here are a few examples. First, the epidemiological findings from the atomic bomb survivors and their children constitute the majority of evidence for the effects of radiation in recent reports from the United Nations Scientific Committee on the Effects of Atomic Radiation (UNSCEAR) in 1994, 2000, 2006, and 2013.^64^ Next, the International Commission on Radiological Protection (ICRP) used radiation risk estimates obtained from the atomic bomb survivors to determine recommendations on radiological protection in the ICRP Publications 60 in 1990 and 103 in 2007.^65^ They cover comprehensive regulation of utilization of radiation in medical, industrial, and daily-life situations (eg, “dose limits” for workers and the public). Domestic laws and regulations for radiological protection follow the ICRP recommendations in many countries. Thus, results from the cohorts of the atomic bomb survivors and their children have been used as the principal source of information for safety measures to protect radiation workers, patients undergoing medical procedures, and the general public.

We sincerely appreciate the loyal participation of the survivors and their children. We will keep in mind that the success of these studies would not have been possible without the cooperation of the victims of the atomic bombings.
